# GBA3 promotes fatty acid oxidation and alleviates non-alcoholic fatty liver by increasing CPT2 transcription

**DOI:** 10.18632/aging.205616

**Published:** 2024-02-29

**Authors:** Juyi Li, Yingqun Ni, Yuanyuan Zhang, Huaizhen Liu

**Affiliations:** 1 Department of Endocrinology, Geriatrics Center, The First Affiliated Hospital of Anhui University of Chinese Medicine, Hefei 230001, Anhui, China; 2 Department of Endocrinology, The First Affiliated Hospital of Anhui University of Chinese Medicine, Hefei 230001, Anhui, China

**Keywords:** non-alcoholic fatty liver disease, GBA3, fatty acid oxidation, necroptosis, oxidative stress

## Abstract

Background: Excessive lipids accumulation and hepatocytes death are prominent characteristics of non-alcoholic fatty liver disease (NAFLD). Nonetheless, the precise pathophysiological mechanisms are not fully elucidated.

Methods: HepG2 cells stimulated with palmitic acids and rats fed with high-fat diet were used as models for NAFLD. The impact of Glucosylceramidase Beta 3 (GBA3) on fatty acid oxidation (FAO) was assessed using Seahorse metabolic analyzer. Lipid content was measured both *in vitro* and *in vivo*. To evaluate NAFLD progression, histological analysis was performed along with measurements of inflammatory factors and liver enzyme levels. Western blot and immunohistochemistry were employed to examine the activity levels of necroptosis. Flow cytometry and reactive oxygen species (ROS) staining were utilized to assess levels of oxidative stress.

Results: GBA3 promoted FAO and enhanced the mitochondrial membrane potential without affecting glycolysis. These reduced the lipid accumulation. Rats supplemented with GBA3 exhibited lower levels of inflammatory factors and liver enzymes, resulting in a slower progression of NAFLD. GBA3 overexpression reduced ROS and the ratio of cell apoptosis. Phosphorylation level was reduced in the essential mediator, MLKL, implicated in necroptosis. Mechanistically, as a transcriptional coactivator, GBA3 promoted the expression of Carnitine Palmitoyltransferase 2 (CPT2), which resulted in enhanced FAO.

Conclusions: Increased FAO resulting from GBA3 reduced oxidative stress and the production of ROS, thereby inhibiting necroptosis and delaying the progression of NAFLD. Our research offers novel insights into the potential therapeutic applications of GBA3 and FAO in the management and treatment of NAFLD.

## INTRODUCTION

Non-alcoholic fatty liver disease (NAFLD) is a prevalent and widespread chronic liver disorder on a global scale [1, 2]. It is estimated that the prevalence of NAFLD has reached 25%, even higher in certain regions [3]. As a consequence of such a high prevalence, NAFLD is expected to become a major cause of liver transplantation and liver cancer [4]. Although still controversial in clinical practice, there has been a proposal to rename NAFLD as metabolic fatty liver disease [5]. As the main hepatic manifestation of metabolic syndrome, NAFLD exhibits a broad spectrum of histological changes, ranging from mild fatty degeneration to more aggressive inflammation and fibrosis. The accumulation of lipids resulting from an imbalance between lipid intake and disposal serves as the starting point for this cascade of pathological events. Elevated intracellular levels of free fatty acids (FFAs), especially saturated fatty acids, induce lipotoxicity and cell death [6]. Cell death represents the endpoint of hepatocyte fate. Subsequent inflammation and loss of hepatic parenchyma drive disease progression. Increasing evidence suggests that the pattern of hepatocyte death during NAFLD involves necroptosis. NAFLD is one of the few human diseases that activate necroptosis without inhibiting apoptosis [7]. The core molecules mediating necroptosis have been shown to play a role in NAFLD [8]. However, the molecular basis of lipid metabolism driving hepatocyte necroptosis remains unclear.

Glucosylceramidase Beta 3 (GBA3), also known as beta-glucosidase, exhibits broad substrate specificity and hydrolyzes many plant β-glycosides in the human diet [9]. GBA3 may play an important role in the interpretation or biotransformation of glycosides [10, 11]. Due to GBA3’s metabolic activity, its expression in the liver shows relatively high levels [12]. Research has found that decreased expression of GBA3 is associated with poor prognosis in hepatocellular carcinoma [13]. However, the role of GBA3 in NAFLD is still unknown.

In this study, we identified GBA3 as a core gene associated with impaired fatty acid oxidation (FAO) in NAFLD through bioinformatics analysis. Further research revealed that GBA3 enhanced FAO by promoting the transcription of Carnitine Palmitoyltransferase 2 (CPT2). Increased FAO reduced lipid accumulation and levels of reactive oxygen species (ROS) in the cells, protecting hepatocytes from necroptosis and improving NAFLD. These findings demonstrate how lipid homeostasis is regulated through the GBA3/CPT2 signaling pathway during the progression of NAFLD. This may provide a potential strategy for the treatment of NAFLD.

## MATERIALS AND METHODS

### Acquisition of FAO-related differentially expressed genes (FRDEGs)

The microarray expression data (GSE160016) were downloaded from the Gene Expression Omnibus. The dataset contained liver transcriptome data from five NAFLD donors and six non-NAFLD donors [14]. When multiple probes were associated with the same gene symbol, the mean value was chosen as the representative expression value for that particular gene.

The “limma” R package with |log fold change| > 2 and screening criteria of P < 0.05 was used to obtain differentially expressed genes (DEGs) [15]. Genes related to FAO were obtained from the GeneCards website [16]. FRDEGs were defined as the genes that overlapped between the set of DEGs and the set of FAO-related genes.

Enrichment analysis was conducted using the Kyoto Encyclopedia of Genes and Genomes (KEGG) and Gene Ontology (GO) databases to gain insight into the signaling pathways and biological functions of FRDEGs.

### Screening of hub FRDEGs

In GSE160016, the FAO activity score was determined by gene set variation analysis [17]. The preset gene set used was REACTOME_MITOCHONDRIAL_FATTY_ACID_BETA_OXIDATION.v2023.1.Hs.gmt [18]. Pearson correlation coefficients were calculated between the expression levels of FRDEGs and the FAO activity score. A receiver operating characteristic curve was employed to explore the potential diagnostic value of the FRDEGs for NAFLD. The mRNA expression levels of hub genes were measured using qRT-PCR in cell and rat models of NAFLD.

### Animals

Male Sprague-Dawley rats aged 4 weeks were obtained from Vital River (Beijing, China). The rats were fed a high-fat diet (60 kcal % fat) for 16 weeks to induce NAFLD [19]. Rats were subjected to adenoviral transduction by administering 1 x 10^11^ viral copies per rat through tail vein injection. We used etomoxir (1 mg/kg, intraperitoneal, every 2 days) to inhibit FAO [20].

Animals were housed in specific pathogen-free environments with controlled lighting (12 hours light and 12 hours dark cycles), temperature (24±2° C), and humidity (50±10%). Animals had unrestricted access to food and water throughout the experiment. Rats were euthanized by administering excessive isoflurane inhalation before liver harvesting.

### Cell culture and transfection

The HepG2 cell line was obtained from Procell Biotechnology Co., Ltd. (Wuhan, China). The cells were cultured in MEM supplemented with 10% fetal bovine serum (v/v), penicillin G (100 units/mL), and streptomycin (100 μg/mL). The cell culture was maintained at 37° C in a humidified atmosphere with 5% CO_2_. A NAFLD cell model was established using palmitic acid (PA) according to previous research [21].

Lentivirus containing pLVX-GBA3-Puro and pLVX-Puro (vector) was purchased from Genechem (Shanghai, China). Stably transfected cells were selected with puromycin and then validated using qRT-PCR and Western blotting.

SiRNA targeting EP300 was purchased from GenePharma (Suzhou, China). Analysis of knockdown efficiency and other experiments were conducted 48 h post-transfection.

### Western blot and co-immunoprecipitation

Cells were prepared with RIPA buffer (Cell Signaling Technology, USA) or Pierce IP lysis buffer (Thermo Fisher Scientific, USA). According to previous study, Western blot and co-immunoprecipitation were performed [22, 23]. In brief, the lysed cells were collected and then centrifuged at 14,000 × g to eliminate cell debris. Protein concentrations were quantified using the BCA Protein Assay Kit (P0009, Beyotime, China). Each sample, containing 10 μg of total protein, was subjected to separation by SDS-PAGE gel and then transferred onto PVDF membranes. The PVDF membrane was incubated with the primary antibody overnight at 4° C. Then, the PVDF membranes were incubated with peroxidase conjugated avidin goat anti-rabbit IgG or goat anti-mouse IgG (1:5000) for one hour at room temperature. The membranes were subsequently scanned, and the protein levels were normalized to β-actin (1:1000) as a control. The Tanon-5200 chemiluminescence imaging system (Shanghai, China) and ImageJ software (NIH, USA) were utilized for recording and quantifying signal intensities. For co-immunoprecipitation, the extract was incubated with the corresponding antibody overnight at 4° C. Protein A&G beads (Bersinbio, Guangzhou, China) were then added and incubated at 4° C for 4 h. The coprecipitated proteins were then washed with SDS loading buffer for 5 min at 95° C. The subsequent results were obtained through Western blotting as previously described. All antibodies were listed in Supplementary Table 1.

### Immunofluorescence and PCR

For immunofluorescence, all procedures were performed as previously reported [24]. The results were visualized by Zeiss laser confocal microscope 900.

Cells or tissues were lysed using TRIzol reagent (Life Technologies, USA). Based on previous study, PCR was performed to determine mRNA expression [21]. The cDNA was synthesized using the ReverTra Ace qPCR RT Kit (Toyobo, Osaka, Japan) and reverse transcription was conducted on a MasterCycler (Eppendorf, GRE, Hamburg, Germany). Subsequently, quantitative real-time PCR (qRT-PCR) was carried out on a LightCycler 480 System (Roche, CH, Basel, Switzerland) with the LightCycler 480 software 1.5.1.62 SP3. B-actin was set as control. The relative mRNA expression was determined using the 2^–ΔΔ^Ct method. The primer sequences can be found in Supplementary Table 2.

### Cell metabolic analysis

The Seahorse XF Glycolytic Rate Assay Kit was utilized to detect glycolysis in cells, as reported in published literature [25]. To measure FAO through oxygen consumption (OCR) in cells, the Seahorse XF Palmitate Oxidation Stress Test Kit was employed.

The mitochondrial membrane potential (Δψm) was measured using JC-10 (Solarbio, Beijing, China). The relative content of tissue and intracellular triglycerides and cholesterol was quantified using commercial kits (Applygen, Beijing, China). The relative content of FFA was measured using a colorimetric assay kit (Elabscience, Wuhan, China). Intracellular lipid droplets were visualized using BODIPY 493/503 (MedChemExpress, USA) and Zeiss laser confocal microscope 900 [26].

### Histological analysis

Livers were sectioned. H&E and Oil Red O stainings were performed using a commercial kit (Solarbio, China). In the IHC process, deparaffinized tissue sections were initially treated with 3% H_2_O_2_ for 15 minutes, followed by a 10-minute incubation in 5% BSA. Subsequently, the sections were subjected to overnight incubation at 4° C with primary antibodies. This was followed by incubation with biotin-conjugated secondary antibodies and visualization using the streptavidin-biotin staining technique. In the case of inadequate staining due to technical issues, samples were excluded.

The relative levels of tumor necrosis factor-a (TNF-α), interleukin 1β (IL1β), and interleukin 10 (IL10) were measured by enzyme-linked immunosorbent assay (ELISA) (R&D Systems, USA).

The relative activities of aminotransferase (ALT), aspartate aminotransferase (AST) and lactate dehydrogenase (LDH) in the liver were measured using commercial kits (Jiancheng, China).

### Cell death and viability assays

A density of 1 × 10^3^ cells/well was used to seed cells in a 96-well plate for CCK-8 assays. At specific timepoints, 10 μl of CCK-8 reagent was added. The optical density (OD) was measured at 450 nm after incubating at 37° C for one hour. The fraction of DNA-replicating cells, representing cell proliferation status, was assessed using the EdU Detection Kit (RiboBio, Guangzhou, China).

Apoptosis was assessed using the Annexin V-FITC Apoptosis Detection Kit (Vazyme, China) along with flow cytometry. Cells located in the upper right quadrants (positive for PI and Annexin V) were classified as necrotic. Relative activity of caspase 8 was determined using the Caspase 8 Activity Assay Kit (Beyotime, China).

### Oxidative stress determination

Tissue or cellular levels of glutathione (GSH) and oxidized glutathione disulfide (GSSG) were quantified by employing commercial kits (Beyotime, China).

Intracellular levels of ROS were measured using a commercial kit (Beyotime, China) and flow cytometry, as well as Zeiss Axio Vert A1 [27]. The hepatic ROS content was quantitatively determined using a commercial kit (Bestbio, China).

### Chromatin immunoprecipitation (ChIP)

To investigate the interaction between EP300 and CPT2, Sonication ChIP Kit (RK20258, ABclonal, USA) was used according to the manufacturer’s procedure in our study.

### Statistical analysis

All analyses were performed in R language (version 4.2.3). All data represented mean ± SEM from three or more experiments. Group differences were assessed using either a 2-tailed Student’s t-test or a Mann-Whitney U test (both 2-tailed). Statistical significance was set at P < 0.05. Commercial kits used in this study were listed entirely in Supplementary Table 3.

### Data sharing statement

The data generated during and/or analyzed during the current study are available from the corresponding author on reasonable request.

## RESULTS

### FRDEGs are involved in NAFLD progression

**Figure 1:**
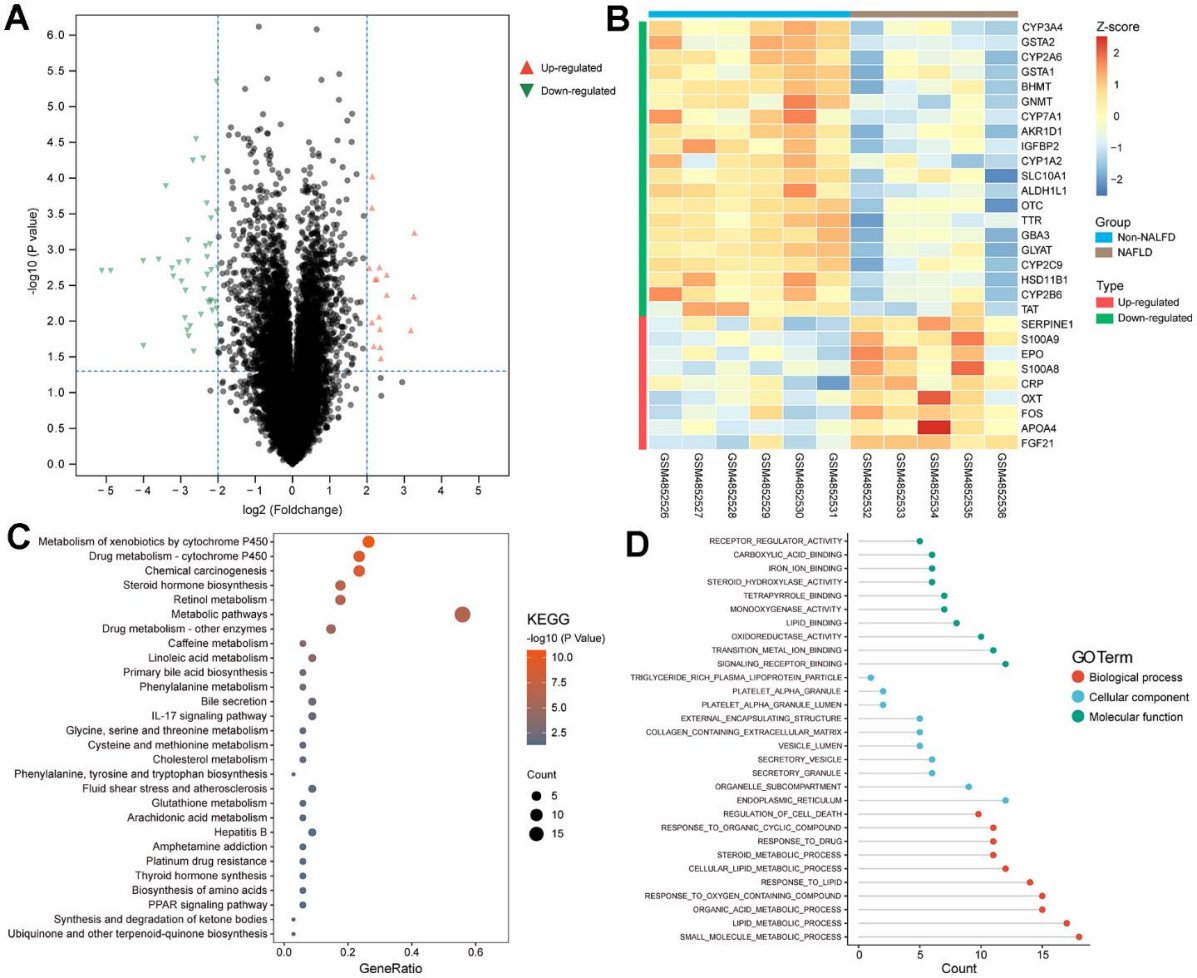
**Screening of FRDEGs from NAFLD.** (**A**) Volcano map of DEGs. (**B**) Heat plot showed the normalized expression profile of 29 FRDEGs obtained from the overlap of DEGs in NAFLD and fatty acid oxidation related genes from GeneCards. (**C**, **D**) Functional enrichment analysis of FRDEGs in KEGG and GO.

We identified 41 downregulated genes and 17 upregulated genes from our analysis (Figure 1A). Out of these, 29 genes are associated with FAO. The expression patterns of these genes are depicted in Figure 1B.

Enrichment analysis unveiled the correlation between FRDEGs and diverse metabolic substances, such as linoleic acid and amino acids (Figure 1C). Additionally, FRDEGs play a role in the regulation of inflammation (such as IL17 signaling pathway) and cell death (Figure 1D). This implies that the FAO may impact inflammation and cell death through specific pathways in NAFLD.

### GBA3 expression is significantly reduced in NAFLD

**Figure 2:**
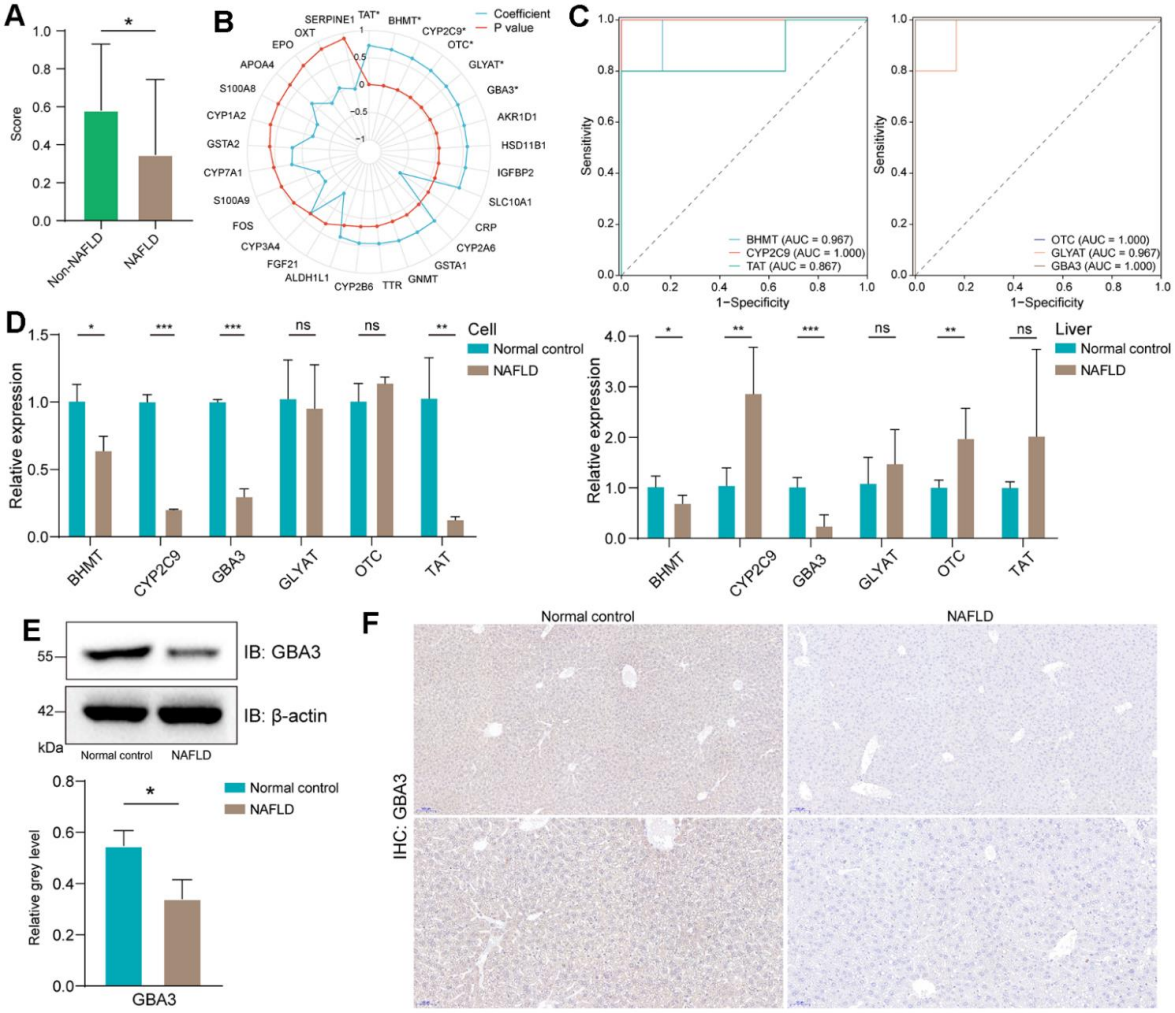
**Identification of GBA3 as a hub gene.** (**A**) FAO scores in GSE160016, P-value was calculated by Mann-Whitney U test (*, P < 0.05). (**B**) Pearson correlation coefficient between the expression of FRDEGs and FAO score (*, P < 0.05). (**C**) The receiver operating characteristic curves of six statistically significant genes in GSE160016. (**D**) Relative mRNA expression of six genes in cells and livers. P-value was calculated by 2-tailed Student’s t test (*, P < 0.05; **, P < 0.01; ***, P < 0.001; ns, not significant). (**E**) Immunoblotting detection of GBA3 in cells. Protein expression was normalized to actin levels and shown as relative values. P-value was calculated by 2-tailed Student’s t test (*, P < 0.05). (**F**) Immunohistochemical images of GBA3 in livers.

First, we calculated the FAO activity score. Consistent with previous studies [28], the activity of FAO was reduced in NAFLD (Figure 2A). This led us to focus on the downregulated FRDEGs that were positively correlated with FAO activity score.

Through Pearson test, we identified six genes that were significant (Figure 2B). Receiver operating characteristic curves demonstrated that these six genes could potentially serve as biomarkers for NAFLD (Figure 2C). To determine the core genes, we used PCR to assess the expression levels of these six genes in the NAFLD model (Figure 2D). The data showed that GBA3 mRNA levels were significantly decreased both *in vivo* and *in vitro*. Therefore, further investigations were conducted. Western blotting and immunohistochemistry revealed that the protein levels of GBA3 were also significantly reduced in NAFLD (Figure 2E, 2F).

### GBA3 facilitates FAO and decreases lipid accumulation

**Figure 3:**
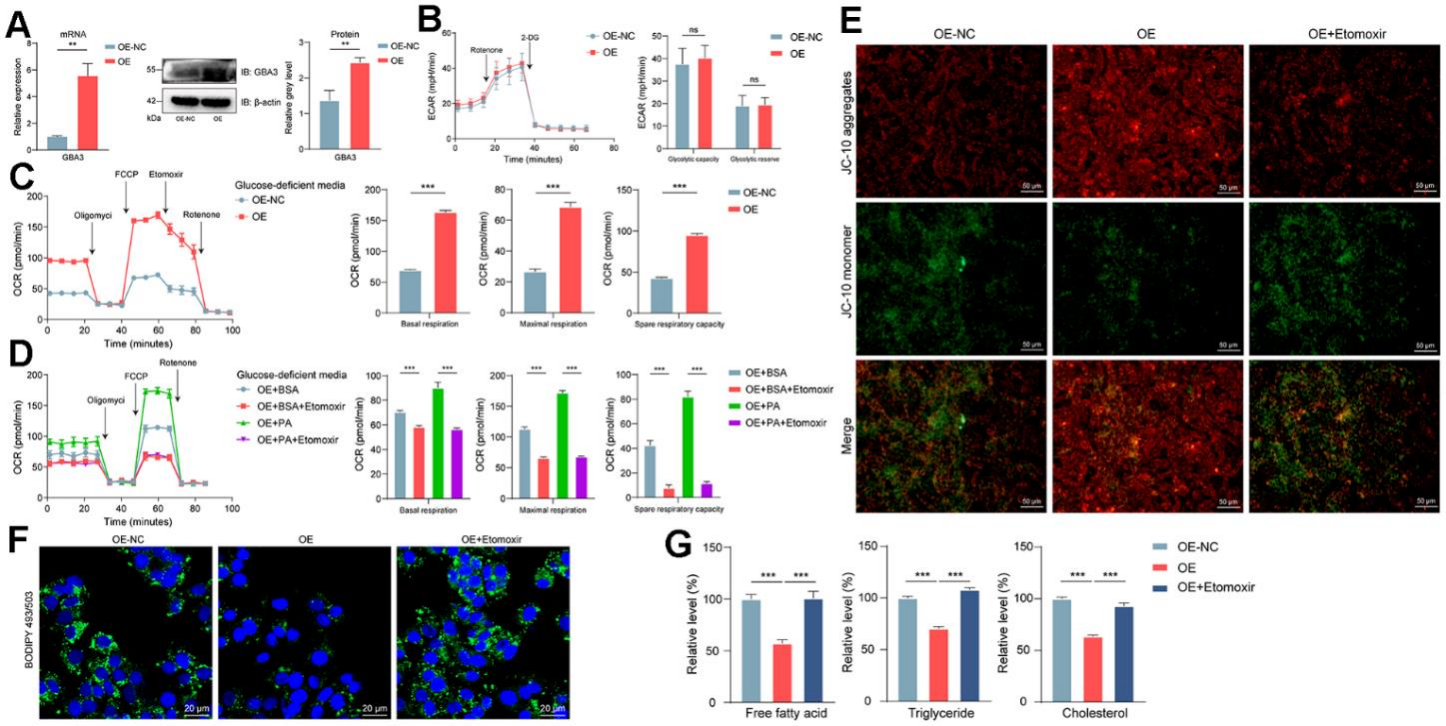
**GBA3 enhanced FAO and reduced the intracellular accumulation of lipids.** (**A**) Constructing the cell line overexpressing GBA3. P-value was calculated by 2-tailed Student’s t test (*, P < 0.01). (**B**) Extracellular acidification rate (ECAR) was analyzed, P-value was calculated by 2-tailed Student’s t test (ns, not significant). (**C**, **D**) Respiratory rates expressed as oxygen consumption rate (OCR) were measured using Seahorse metabolic analyzer. P-value was calculated by 2-tailed Student’s t test (***, P < 0.001). (**E**) Representative images of JC-10 staining (Scale bars, 50 μm). (**F**) Lipid droplet was visualized by Bodipy 493/503 (green fluorescence), nuclei were counterstained blue with DAPI (blue fluorescence) (Scale bars, 20 μm). (**G**) Relative levels of free fatty acid, triglyceride and cholesterol were quantified in cells, P-value was calculated by 2-tailed Student’s t test (***, P < 0.001).

First, we stably overexpressed GBA3 in cells (Figure 3A). Previous studies have shown that GBA3 can hydrolyze various substrates, including β-glucose linked to hydrophobic groups [29]. This led us to explore its relationship with sugar metabolism before discussing the role of GBA3 in FAO. The data indicated that GBA3 had no effect on extracellular acidification rate, neither in glycolytic capacity nor in reserves (Figure 3B). In the absence of glucose, we found that GBA3 promoted FAO. The mitochondrial oxygen consumption rate significantly increased (Figure 3C). After supplementing with exogenous PA as a substrate, mitochondrial respiration capacity further enhanced (Figure 3D). The improvement in FAO led to an increase in JC10 aggregates (Figure 3E), suggesting a significant elevation in mitochondrial membrane potential, resulting in more energy production.

Accumulation of lipids is one of the initiating factors of NAFLD. Images from confocal microscopy showed that GBA3 reduced lipids in cells (Figure 3F). As shown in Figure 3G, GBA3 led to a significant decrease in cellular content of FFA, triglycerides, and cholesterol. However, the effect of GBA3 was blocked by etomoxir. This indicates that the decrease in lipid content in cells is caused by the increased demand for substrates due to the higher FAO induced by GBA3.

### GBA3 protects rats from NAFLD

**Figure 4:**
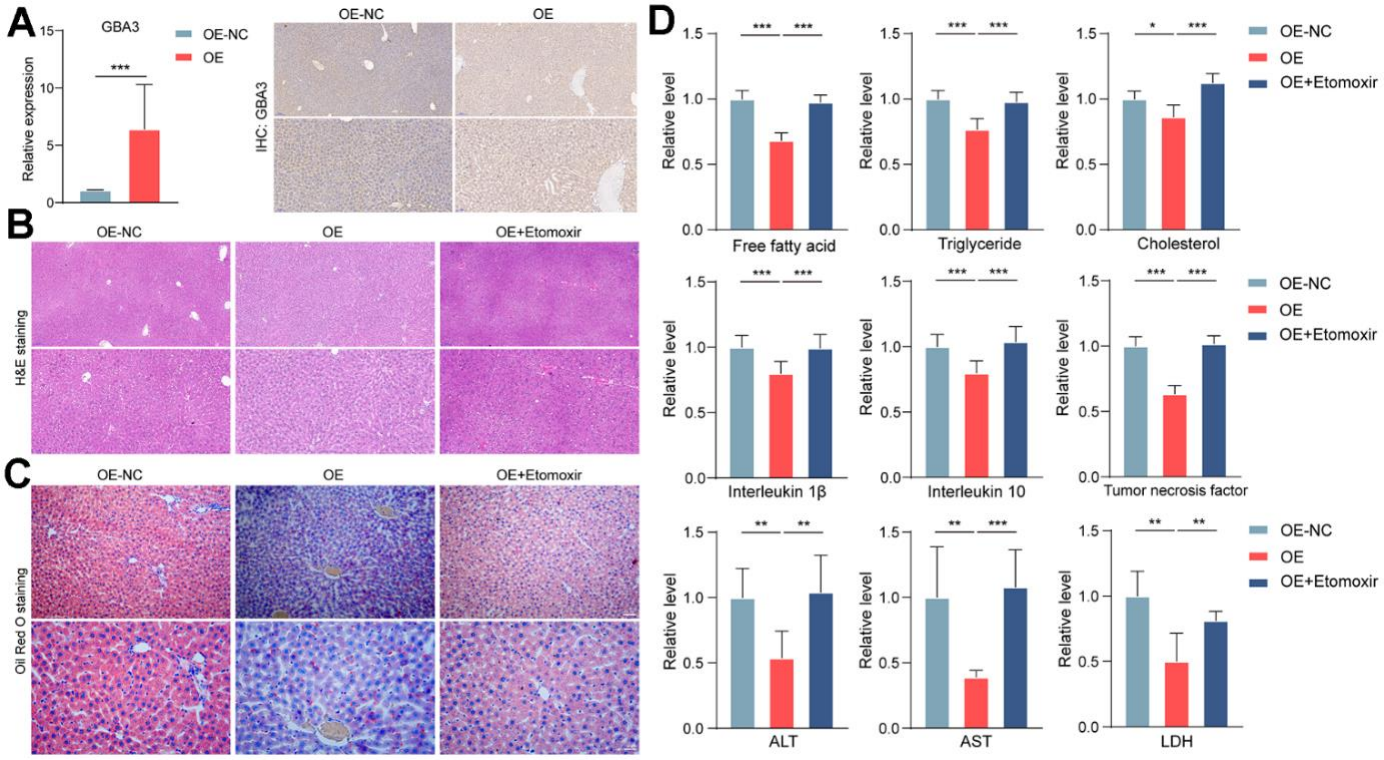
**GBA3 protected rats from NAFLD.** (**A**) Successful supplementation of GBA3 in the liver of mice was confirmed through PCR and immunohistochemistry. P-value was calculated by 2-tailed Student’s t test (***, P < 0.001). (**B**) H&E staining of livers. (**C**) Oil Red O staining was used to assess lipid content. (**D**) Relative levels of free fatty acid, triglyceride, cholesterol, inflammatory factors, ALT, AST and LDH were detected in livers, P-value was calculated by 2-tailed Student’s t test (*, P < 0.05; **, P < 0.01; ***, P < 0.001).

So far, we have identified the crucial role of GBA3 in counteracting lipid overload by stimulating FAO *in vitro*. To determine the physiological function of GBA3, we transferred it to a NAFLD rat model. Before being fed a high-fat diet, the rats received adenovirus expressing GBA3 (Figure 4A).

Histological analysis showed that GBA3 alleviated hepatic steatosis induced by a high-fat diet (Figure 4B, 4C). Compared to the control group, GBA3 reduced lipid accumulation. Additionally, levels of inflammatory factors and transaminases in the liver were significantly decreased. Etomoxir partially reversed the effect of GBA3 (Figure 4D). These data support the physiological protective role of GBA3 in high-fat diet-induced NAFLD.

### GBA3 protects cells from necroptosis by reducing ROS levels

**Figure 5:**
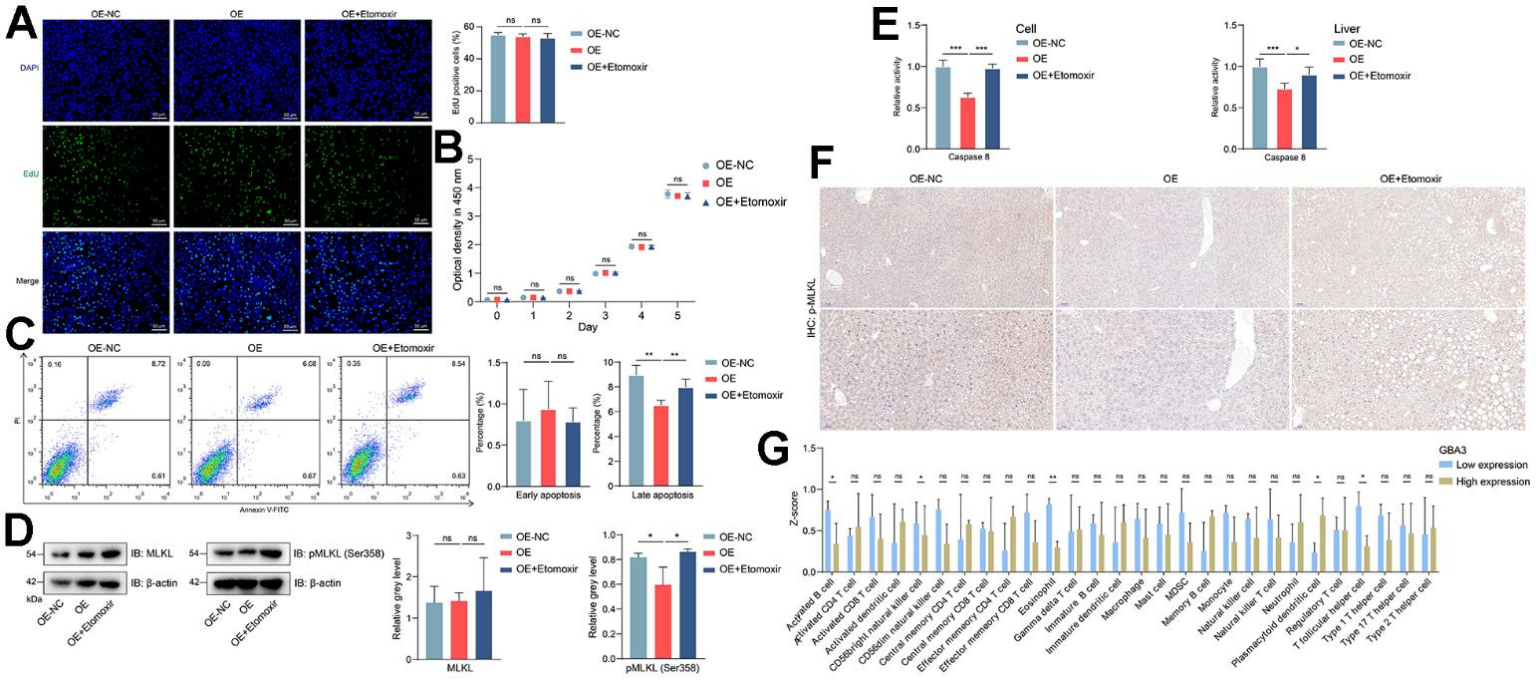
**GBA3 reduced the occurrence of necroptosis.** (**A**) EdU staining (green fluorescence) on cell proliferation, nuclei were counterstained blue with DAPI (Scale bars, 50 μm). (**B**) Cell proliferation was detected by measuring the optical density at 450 nm, P-value was calculated by 2-tailed Student’s t test (ns, not significant). (**C**) Percentage of apoptotic cell was assessed via flow cytometry, P-value was calculated by 2-tailed Student’s t test (ns, not significant; **, P < 0.01). (**D**) Immunoblotting detection of MLKL and pMLKL (Ser358) in cells. Protein expression was normalized to actin levels and shown as relative values. P-value was calculated by 2-tailed Student’s t test (*, P < 0.05). (**E**) The activity of caspase 8 was assessed by measuring the optical density at 405 nm. Incremental activity of caspase 8 was expressed as a percentage of control, P-value was calculated by 2-tailed Student’s t test (*, P < 0.05; ***, P < 0.001). (**F**) Phosphorylation level of MLKL in livers detected by immunohistochemistry. (**G**) The infiltrated level of immune cells, P-value was calculated by Mann-Whitney U test (*, P < 0.05; **, P < 0.01).

Apoptosis of hepatocytes caused by lipid overload often promotes the development of NAFLD [30]. Therefore, we investigated whether the pro-FAO effect of GBA3 is beneficial for cell survival. CCK8 and EdU staining showed that GBA3 had no effect on cell proliferation (Figure 5A, 5B). We then turned our attention to apoptosis. Data from flow cytometry showed that GBA3 overexpression only reduced the percentage of cells in the upper right quadrant (PI positive and Annexin V positive) (Figure 5C). This indicates that the protective effect of GBA3 mainly prevents cells from undergoing late-stage apoptosis.

Necroptosis is a late-stage apoptosis that induces inflammation and infiltration of immune cells [31]. MLKL is the core mediator of necroptosis. Its phosphorylation is a specific and irreversible molecular event that drives necroptosis [32]. Consequently, we performed related experiments. As expected, GBA3 overexpression reduced the phosphorylation level of MLKL and the activity of Caspase 8 (Figure 5D, 5E). Similar trends were observed *in vivo* (Figure 5F). We divided all NAFLD samples from GSE160016 into high-expression and low-expression groups based on GBA3 expression level. Then, we compared the levels of immune cell infiltration between the two groups. The data showed that when GBA3 was highly expressed, the infiltration levels of activated B cells, natural killer cells, eosinophils, and T follicular helper cells decreased (Figure 5G). Most of the other changes in immune cells were not significant. These findings suggest that GBA3 can inhibit the transition of cells to necroptosis and prevent the occurrence of inflammation.

**Figure 6:**
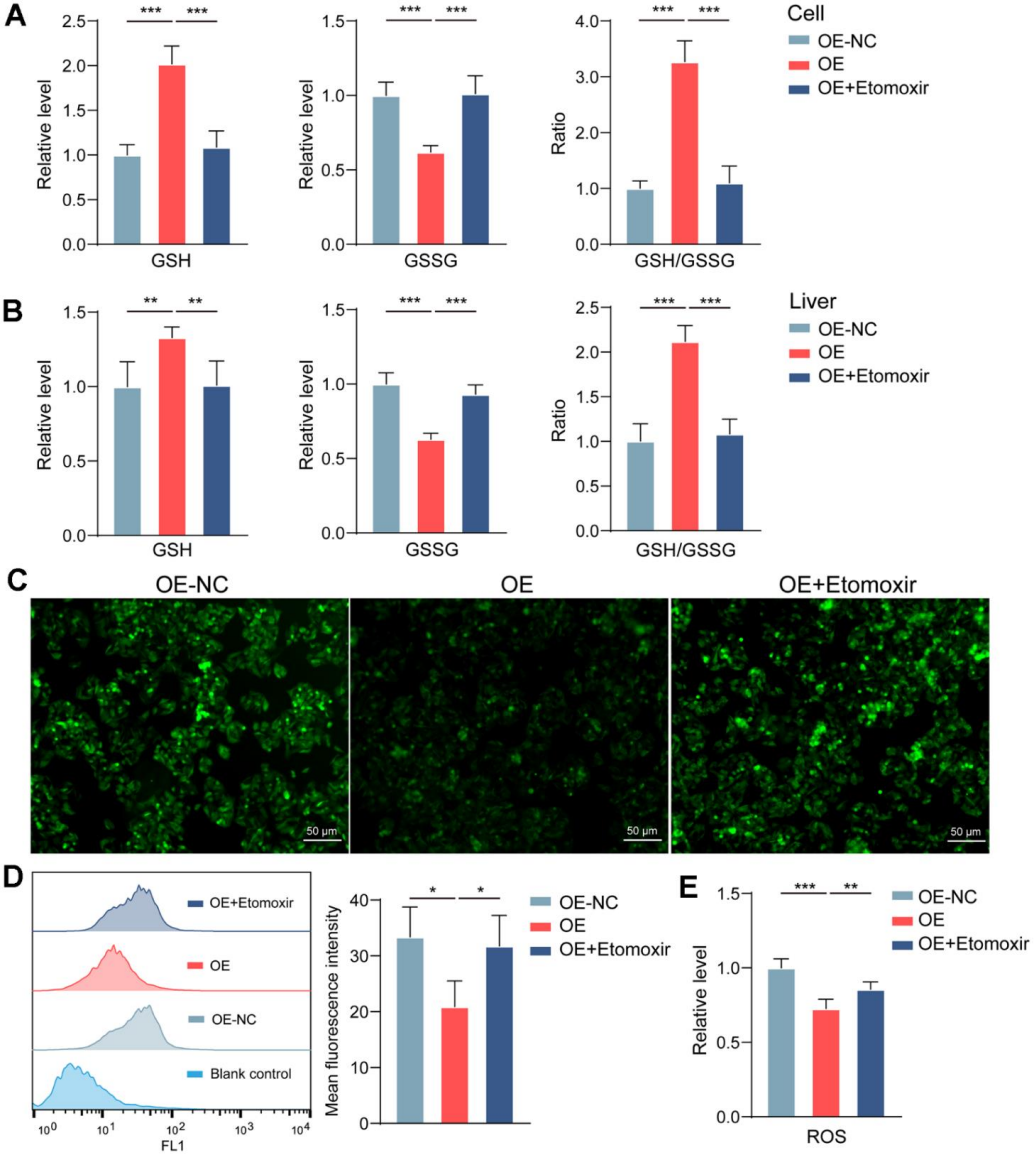
**GBA3 maintains mitochondrial homeostasis by reducing ROS.** (**A**, **B**) Levels of GSH and GSSG were quantified (**, P < 0.01; ***, P < 0.001). (**C**) Representative image of ROS detection in cells (Scale bars, 50 μm). (**D**) ROS was quantified via flow cytometry, P-value was calculated by 2-tailed Student’s t test (*, P < 0.05). (**E**) Hepatic ROS content was quantitatively determined, P-value was calculated by 2-tailed Student’s t test (**, P < 0.01; ***, P < 0.001).

However, it is still unclear how increased FAO protects cells from necroptosis. Some studies have drawn our attention to this. Robert O Watson et al. found that excess ROS in mitochondria can trigger necrotic apoptosis [33]. Enhanced FAO promoted the generation of reducing substances and reduced ROS production [34]. In this study, we found that GBA3 increased the GSH/GSSG ratio (Figure 6A, 6B) and reduced ROS levels (Figure 6C–6E), both *in vitro* and *in vivo*. In conclusion, the above data suggest that GBA3 protects cells from necroptosis by increasing FAO and reducing ROS levels.

### GBA3 upregulates the expression of CPT2 to promote FAO

**Figure 7:**
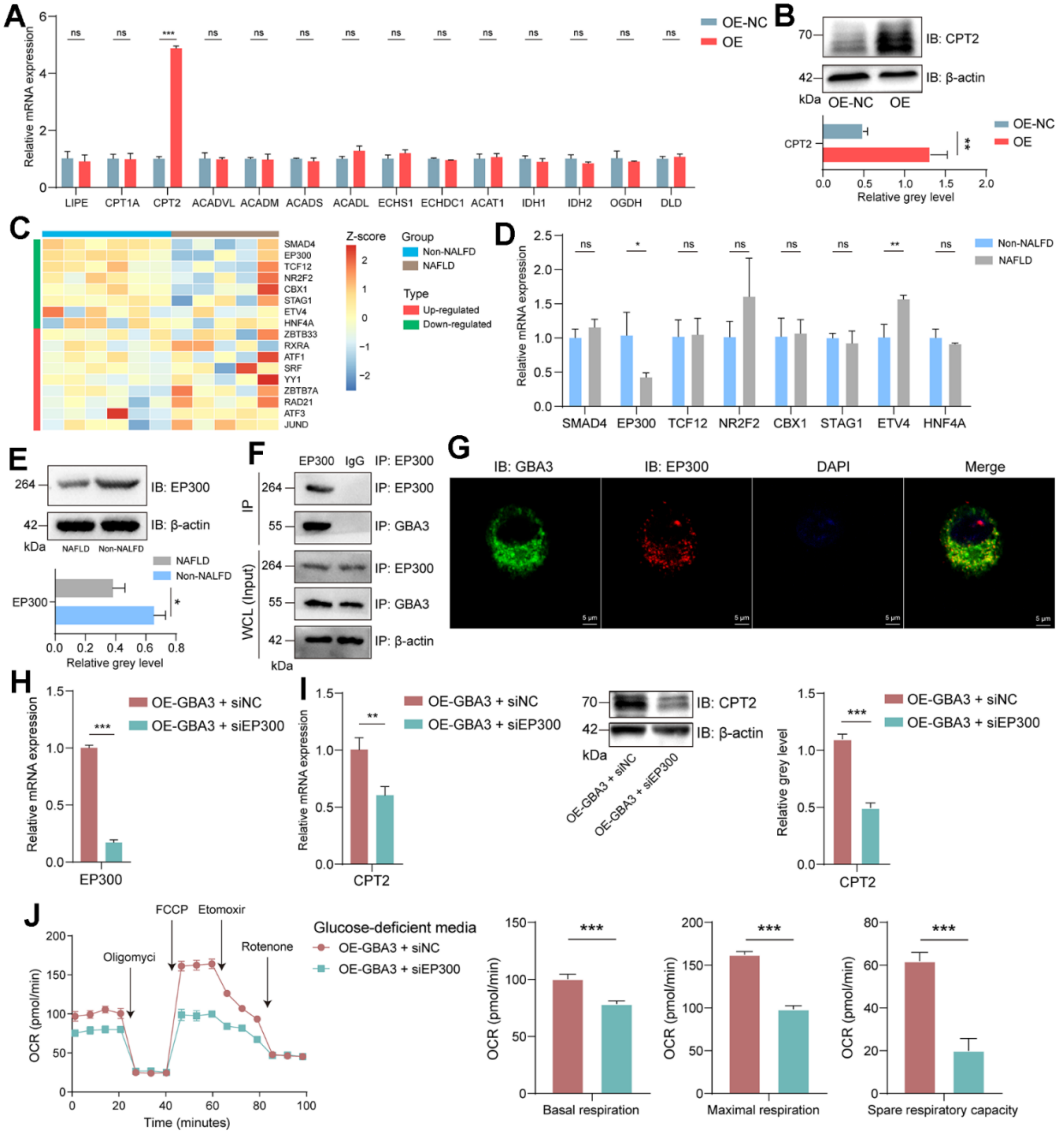
**GBA3 interacted with EP300 to promote CPT2 transcription.** (**A**) Relative mRNA expression, P-value was calculated by 2-tailed Student’s t test (***, P < 0.001; ns, not significant). (**B**) Immunoblotting detection of CPT2. P-value was calculated by 2-tailed Student’s t test (*, P < 0.05). (**C**) Heat plot showed the normalized expression profile of transcription factors of CPT2 in GSE160016. (**D**) Relative mRNA expression of 8 transcription factors, P-value was calculated by 2-tailed Student’s t test (*, P < 0.05; **, P < 0.01; ns, not significant). (**E**) Immunoblotting detection of EP300. P-value was calculated by 2-tailed Student’s t test (*, P < 0.05). (**F**) Coimmunoprecipitation of GBA3 with endogenous EP300. (**G**) Representative images of immunofluorescence staining of GBA3 and EP300. Nuclei were counterstained blue with DAPI (Scale bars, 5 μm). (**H**) Knockdown of EP300 in cells overexpressing GBA3. (**I**) CPT2 expression was confirmed through PCR and Western blot. P-value was calculated by 2-tailed Student’s t test (***, P < 0.001). (**J**) Respiratory rates expressed as oxygen consumption rate (OCR) were measured using Seahorse metabolic analyzer. P-value was calculated by 2-tailed Student’s t test (***, P < 0.001).

Finally, we focus on the specific mechanism of GBA3 in regulating FAO. FAO is regulated by a series of enzymes. PCR was used to preliminarily detect the expression levels of these enzymes. As shown in Figure 7A, the transcription level of CPT2 increased. WB showed an increase in the expression of CPT2 (Figure 7B). This indicates that CPT2 is involved in GBA3-mediated metabolic reprogramming. We obtained the transcription factors of CPT2 from the Cistrome Data Browser. Their expression in GSE160016 is shown in Figure 7C. As the expression of GBA3 is downregulated in NAFLD, we paid more attention to the transcription factors whose expression levels also decreased. PCR showed that only the expression of EP300 was decreased in the NAFLD cell model (Figure 7D). The results of WB further confirmed this finding (Figure 7E). As shown in Supplementary Figure 1, we demonstrated EP300 was transcription factor of CPT2 via ChIP. We infer the existence of interaction between GBA3 and EP300. Immunoprecipitation (Figure 7F) and immunofluorescence (Figure 7G) demonstrated our hypothesis.

We used siRNA to interfere with the expression of EP300 to investigate whether EP300 is involved in GBA3-mediated regulation of FAO (Figure 7H). The data showed that when the expression of EP300 was knocked down, the expression of CPT2 decreased (Figure 7I). The promoting effect of GBA3 on FAO was also blocked (Figure 7J). All these data indicate that GBA3 enhances FAO by interacting with EP300 and promoting the transcription of CPT2.

## DISCUSSION

Our study provides the first evidence of the crucial contribution of GBA3 in the pathology of NAFLD in liver cells. The data suggest that GBA3 promotes the transcription of CPT2, thus alleviating NAFLD by promoting FAO. Our study provides evidence supporting the toxic effects of fatty acid overload on liver cells [35]. Furthermore, it further indicates that the regulation of lipid metabolism is a potential strategy for managing NAFLD.

Imbalance in FFA metabolism is a key event leading to the occurrence of NAFLD [36]. In the highly reversible stage of simple steatosis, various cellular stress responses are activated to accelerate FFA processing, thus counteracting the progression of non-alcoholic fatty liver [37, 38]. Enhancing FAO in liver cells can greatly help the liver process excess lipids [39]. Consistent with these studies, our work suggests that GBA3 can enhance cytoplasmic FAO, thereby counteracting the pathological changes caused by NAFLD. If the development of NAFLD exceeds the functional adaptability of mitochondria, FAO may collapse, leading to the excessive production of lipotoxic metabolites and reactive oxygen species [40]. This will activate signaling pathways of inflammation, leading to hepatocyte apoptosis [41]. In fact, oxidative DNA damage has been detected in the livers of late-stage NAFLD patients [42].

In addition to FFA overload, hepatocyte necroptosis is also a key event in the process of NAFLD [43]. Necrosis and necroinflammation are typical histological manifestations of NAFLD [44]. Necroptosis simultaneously exhibits morphological and biochemical characteristics of both apoptosis and necrosis. Molecules that promote apoptosis, such as RIPK1, can also mediate necroptosis. Sanjoy Roychowdhury et al. found that the occurrence of necroptosis in hepatocytes may not require the activity of RIPK1. The role of RIPK3, another key molecule in necroptosis, in NAFLD remains controversial [45, 46]. The work of Stefan Wirtz et al. suggests that MLKL can mediate necroptosis independently of RIPK3. Furthermore, there is evidence that the loss of Caspase 8 in hepatocytes contributes to the initiation of necroptosis. Therefore, in our work, the combination test of MLKL and Caspase 8 was used to determine the activation of necroptosis. Given the limited knowledge of the association between lipid metabolism and necroptosis in NAFLD, we used cell and animal models to focus on the regulation of necroptosis by FAO. Our results indicate that FAO reduces the level of ROS, maintains mitochondrial homeostasis, and prevents the triggering of necroptosis.

Currently, research on the functions of GBA3 is still in the exploratory stage. In this study, we have demonstrated through *in vitro* experiments that there is an interaction between GBA3 and EP300. This contributes to the promotion of CPT2 by EP300. The non-enzymatic function of GBA3 has been previously discovered. The work of Tadashi Suzuki and others has demonstrated a physical interaction between GBA3 and NEU2 [47]. These phenomena suggest that the function of GBA3 may not be limited to enzymes. Future research may further explore the physiological role of GBA3 from a protein perspective.

## CONCLUSIONS

In summary, this study provides direct evidence for the important role of GBA3 in the pathogenesis of NAFLD. Mouse and *in vitro* experiments demonstrate that GBA3 interacts with EP300 to promote the transcription of CPT2, thereby enhancing FAO. The resulting reduced ROS levels prevent necrotic apoptosis from being triggered. Our research supports the potential therapeutic value of GBA3 in NAFLD.

## Supplementary Material

Supplementary Figure 1

Supplementary Tables
