# Supplementary figures and images for: GBA3 promotes fatty acid oxidation and alleviates non-alcoholic fatty liver by increasing CPT2 transcription
Source: Aging (Albany NY). 2024 Feb 29;16(5):4591–608. doi: 10.18632/aging.205616 (PMC10968678; doi:10.18632/aging.205616)

## SUPPLEMENTARY FIGURE

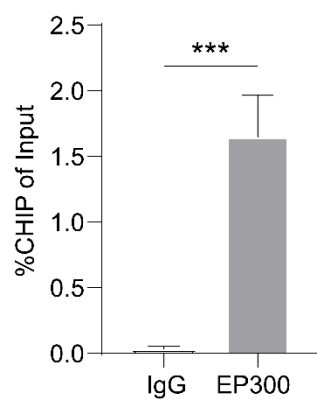

Supplementary Figure 1. Chromatin immunoprecipitation assay results.

Supplement: Supplementary Figure 1 [file aging-16-205616-s001.pdf]
