# Supplementary material for: GBA3 promotes fatty acid oxidation and alleviates non-alcoholic fatty liver by increasing CPT2 transcription
Source: Aging (Albany NY). 2024 Feb 29;16(5):4591–608. doi: 10.18632/aging.205616 (PMC10968678; doi:10.18632/aging.205616)
Supplement: Supplementary Tables [file aging-16-205616-s002.pdf]

SUPPLEMENTARY TABLES

Supplementary Table 1. Antibodies used in the study.

Gene symbol	Reactivity	Dilution	Source	Catalog
GBA3	Human	1: 1000	Abcam	ab154897
	Rat	1: 100	Bioss	bs-13298R
MLKL	Human	1: 1000	Cell Signaling Technology	37705
p-MLKL (S358)	Human	1: 1000	Cell Signaling Technology	18640
	Rat	1: 100	Affinity Biosciences	AF7420
B-actin	Human	1: 1000	Cell Signaling Technology	3700
CPT2	Human	1: 1000	ABclonal Technology	A0653
EP300	Human	1: 1000	Cell Signaling Technology	86377/54062

Supplementary Table 2. Primers used in the study.

Gene symbol	Species		Sequence (5'- 3')
BHMT	Human	Forward	AGCAGGAGGAGTGAGTCAGA
		Reverse	CTGCCACAGGTTTACCGGAT
	Rat	Forward	GGCGAAGTCGTGATCGGAGA
		Reverse	GATGAAGCTGCCGAACTGCC
CYP2C9	Human	Forward	TGGATGAAGGTGGCAATTTT
		Reverse	GGGCTTCTCCCACACAAAT
	Rat	Forward	TCTGCAATACTTTCCCTGCCA
		Reverse	GGCTCTTGTGATGCTGTTCCA
GBA3	Human	Forward	ATGGCTTTCCCTGCAGGATT
		Reverse	CTACAGATGTGCTTCAAGGC
	Rat	Forward	TCACCGAGAATGGGTTTCCC
		Reverse	CCACGCACAGTAGAGTTGGA
GLYAT	Human	Forward	AATGGTGGCAAACCCAAGGC
		Reverse	GCCAGAGTGAATGCAGGGAGA
	Rat	Forward	TCCGCCCTCAAGAACAGGAAA
		Reverse	GCCCAGGAACTCCTGACAGT
OTC	Human	Forward	TGGCTGATTACCTCACGCTC
		Reverse	TTCTTCTGGCTTTCTGGGCAA
	Rat	Forward	TCTTAGCTTGCCAGTGGCCC
		Reverse	GGACTGGCTTCCCATACCGAA
TAT	Human	Forward	AGCATCCTATGTCGCACCCC
		Reverse	AGCCAACGCCCCATAACAGA
	Rat	Forward	CTGCAGTCAGGCCATTGAGC
		Reverse	CCAAGACTTCTCGGGCAGGA
β-Actin	Human	Forward	AGAAAATCTGGCACCACACC
		Reverse	CCATCTCTTGCTCGAAGTCC
	Rat	Forward	AACCTTCTTGCAGCTCCTCCG
		Reverse	CATACCCACCATCACACCCTGG
LIPE	Human	Forward	GTGAGTCCCAGCCAGGTGTG
		Reverse	GTGCACGTCCAGGTTCTGTG
CPT1A	Human	Forward	CTTGCCCTGAGACGGGGATT
		Reverse	TCCATGGTCTCCTCCAAGGC

CPT2	Human	Forward	ACGAGTCCTGTAGCACTGCC
		Reverse	TGCTGAAGCTCACCAGCACT
ACADVL	Human	Forward	AGATTCGGAGATGCAGGCGG
		Reverse	CTGGTCAGAGCGTCAGAGGG
ACADM	Human	Forward	CAAGGCCGTGACCCGTGTAT
		Reverse	CTGCAGCATCGCCCGAAC
ACADS	Human	Forward	GGGCGACTCATGGGTTCTGA
		Reverse	GTTTTGCAGGGCTCTGTCCG
ACADL	Human	Forward	AGCTGATCGTCCTCCCTCCC
		Reverse	GAATGAGAACATCGCGCGGC
ECHS1	Human	Forward	TTCGCCTCGGGTGCTAACTT
		Reverse	TTGGGGCGGTTCAGTTGGAT
ECHDC1	Human	Forward	GGGAGCGGAAACAAAGGCAC
		Reverse	TTTCGCCATTTCGTCGCAGG
ACAT1	Human	Forward	CGGAGGCTGGTGCAGGAAAT
		Reverse	ACCAAGCTTAGTGGCTGGCA
IDH1	Human	Forward	TTCCGGGTGCCTGGAGTTTA
		Reverse	CTACCACAGAACCGCCACTGA
IDH2	Human	Forward	GCCATAGGCTTCCAGCGACT
		Reverse	ACCATCCATCTCCACCACGG
OGDH	Human	Forward	GATGGGCAAGACCAAAGCCG
		Reverse	GCCTTGACGATCTGCCTTGC
DLD	Human	Forward	TGCAGAGCTGGAGTCGTGTG
		Reverse	ATCCTCCAGGACCAGAACCT
SMAD4	Human	Forward	CCGCTGCGGATCAAAATTGC
		Reverse	GCACAATGCTCAGACAGGCATC
EP300	Human	Forward	AGGGATGAATGCGGGCATGA
		Reverse	GGGAGAGCCCTGCTGAAGAG
TCF12	Human	Forward	ACTCCCTTTGCCTGTGTGGA
		Reverse	GCCCATTCCAGGGACAGGAT
NR2F2	Human	Forward	CCTGCAGGCTAGTGCCTACTT
		Reverse	ACCGCTTGCATCTTCCTCCT
CBX1	Human	Forward	TTTGTTCGGCTGAGGGGAGG
		Reverse	AAAGGGTGACGCTGCTCAGA
STAG1	Human	Forward	CACCAGATGGCGGGTTAGGA
		Reverse	CCCCCAAAAGTCTCCGGTGT
ETV4	Human	Forward	AAAACAAGTCGGTGCGCTGG
		Reverse	TTCATCCTCCGCTCCATCCG
HNF4A	Human	Forward	CAGAATGAGCGGGACCGGAT
		Reverse	GCTGGCAATCTTCTTCGCCC

Supplementary Table 3. Commercial kits used in the study.

Name	Source	Catalog
Seahorse XF Glycolytic Rate Assay Kit	Agilent	103344-100
Seahorse XF Palmitate Oxidation Stress Test Kit		103693-100
Triglyceride Content Enzymatic Assay Kit	Applygen	E1013-50
Total Cholesterol Content Enzymatic Assay Kit		E1015-50
Non-esterified Free Fatty Acids Colorimetric Assay Kit	Elabscience	E-BC-K013-M
Hematoxylin-Eosin Stain Kit	Solarbio	G1120
Modified Oil Red O Stain Kit, Environmentally Friendly		G1263
Rat TNF-alpha Quantikine ELISA Kit	R&D Systems	RTA00
Rat IL-1 beta/IL-1F2 Quantikine ELISA Kit		RLB00
Rat IL-10 Quantikine ELISA Kit		R1000
Alanine aminotransferase Assay Kit	Jiancheng	C009-2-1
Aspartate aminotransferase Assay Kit		C010-2-1
Lactate dehydrogenase assay kit		A020-2-2
Cell-Light EdU Apollo488 *In Vitro* Kit	RiboBio	C10310-3
Annexin V-FITC/PI Apoptosis Detection Kit	Vazyme	A211
Caspase 8 Activity Assay Ki	Beyotime	C1151
GSH and GSSG Assay Kit		S0053
Reactive Oxygen Species Assay Kit		S0033S
BCA Protein Assay Kit		P0009
Tissue Reactive Oxygen Species Test Kit	BestBio	BB-470515
Sonication ChIP Kit	ABclonal	RK20258
